# Cerebro-cerebellar connectivity is increased in primary lateral sclerosis

**DOI:** 10.1016/j.nicl.2014.12.009

**Published:** 2014-12-09

**Authors:** Avner Meoded, Arthur E. Morrissette, Rohan Katipally, Olivia Schanz, Stephen J. Gotts, Mary Kay Floeter

**Affiliations:** a National Institute of Neurological Disorders and Stroke, National Institutes of Health, Bethesda, MD, USA; b National Institute of Mental Health, National Institutes of Health, Bethesda, MD, USA

**Keywords:** AFNI, analysis of functional neuroimages, ALS, amyotrophic lateral sclerosis, ALSFRS-R, amyotrophic lateral sclerosis rating scale, ANCOVA, analysis of covariance, BOLD, blood oxygen-level dependent, DTI, diffusion tensor imaging, Epi, echo planar imaging, FA, fractional anisotropy, FWE, family-wise error, fMRI, functional magnetic resonance imaging, FSL, FMRIB Software Library, MNI, Montreal Neurological Institute, PLS, primary lateral sclerosis, ROI, region of interest, TFCE, threshold-free cluster enhancement, TORTOISE, tolerably obsessive registration and tensor optimization indolent software ensemble, TBSS, tract based spatial statistics, Motor neuron disease, Resting state functional MRI, Connectivity, Cerebellum, Primary lateral sclerosis

## Abstract

Increased functional connectivity in resting state networks was found in several studies of patients with motor neuron disorders, although diffusion tensor imaging studies consistently show loss of white matter integrity. To understand the relationship between structural connectivity and functional connectivity, we examined the structural connections between regions with altered functional connectivity in patients with primary lateral sclerosis (PLS), a long-lived motor neuron disease. Connectivity matrices were constructed from resting state fMRI in 16 PLS patients to identify areas of differing connectivity between patients and healthy controls. Probabilistic fiber tracking was used to examine structural connections between regions of differing connectivity. PLS patients had 12 regions with increased functional connectivity compared to controls, with a predominance of cerebro-cerebellar connections. Increased functional connectivity was strongest between the cerebellum and cortical motor areas and between the cerebellum and frontal and temporal cortex. Fiber tracking detected no difference in connections between regions with increased functional connectivity. We conclude that functional connectivity changes are not strongly based in structural connectivity. Increased functional connectivity may be caused by common inputs, or by reduced selectivity of cortical activation, which could result from loss of intracortical inhibition when cortical afferents are intact.

## Highlights

• Functional connectivity is increased in primary lateral sclerosis.
• Functional connections with the cerebellum were prominent.
• Cortico-cerebellar connectivity correlated with clinical measures.
• No corresponding changes occurred in structural connectivity.

## 1 Introduction

Functional connectivity in resting state networks is decreased in many neurodegenerative and neuropsychiatric disorders (Alexander-Bloch et al., 2013; Chhatwal and Sperling, 2012; Damoiseaux et al., 2012; Gotts et al., 2012). In motor neuron disorders such as amyotrophic lateral sclerosis (ALS), some studies report that functional connectivity is increased compared to healthy controls, particularly in the regional sensorimotor network (Douaud et al., 2011; Jelsone-Swain et al., 2010; Verstraete et al., 2010), whereas others report decreased functional connectivity (Agosta et al., 2011; Mohammadi et al., 2009; Schmidt et al., 2014; Zhou et al., 2014). Increased functional connectivity in the sensorimotor network has also been reported in primary lateral sclerosis (PLS), a rare motor neuron disorder variant with slow progression and long survival periods (Agosta et al., 2014).

In motor neuron disorders, functional connectivity may be affected by the stage of disease and the structural integrity of long-range white matter tracts in the brain. In ALS, the most common motor neuron disorder, the earliest stage of degeneration affects motor cortex neurons, with spread of degeneration to anterior cortical areas over time (Braak et al., 2013). Diffusion tensor imaging (DTI) shows disruption of the corticospinal tract and callosal white matter (Agosta et al., 2010; Ciccarelli et al., 2009; Ellis et al., 1999; Iwata et al., 2008). In ALS, functional connectivity was inversely related to the diffusion tensor imaging measures of the structural integrity of cortical white matter tracts (Douaud et al., 2011; Verstraete et al., 2011).

To date, most rs-fMRI studies in motor neuron disorders have examined functional connectivity changes within regional rs-fMRI networks that have been defined in healthy controls. That approach may miss new patterns of connectivity emerging as a consequence of the disease. New functional connectivity patterns may be particularly important in PLS patients because patients' long survival allows an extended period in which neuroplasticity could occur. Some evidence indicates that the relationship between functional connectivity and structural integrity is affected by the pace of disease progression, with less alteration of functional connectivity in ALS patients who have slower rates of disease progression (Douaud et al., 2011). In this study we combined functional and structural approaches to look for changes in connectivity in PLS patients. In this study we used a data-driven approach to allow visualization of differing functional connectivity patterns in PLS patients (Gotts et al., 2012). Diffusion tensor imaging was used to assess the integrity of white matter tracts and structural connections between regions of differing functional connectivity.

## 2 Methods

### 2.1 Subjects

All subjects gave written informed consent for protocols approved by the NIH Combined Neuroscience Institutional Review Board (NCT00015444; NCT01517087). All subjects had examinations by a neurologist, and all healthy controls had normal neurological examinations. The PLS group consisted of successive clinic patients seen during 2012 and 2013 who had no contraindications to MRI scanning. PLS patients were diagnosed by clinical criteria (Pringle et al., 1992), with testing to exclude alternative diagnoses. Evaluations included neurological examinations and interviews with caregivers. Clinical scales used for correlation analyses included the ALSFRS-R (Cedarbaum et al., 1999), mini-mental state examination (Folstein et al., 1975), measures of timed gait, and foot- and finger-tapping speed. None of the patients met criteria for frontotemporal dementia (Rascovsky et al., 2011) nor had a family history of PLS, ALS or frontotemporal dementia.

### 2.2 Imaging acquisition

A 3 T MRI scanner was used with a receive-only, eight-channel head coil (GE Medical Systems, Milwaukee, WI). The sequences acquired in each subject included:

1. A high-resolution T1-weighted sequence (slice thickness 1 mm); 2) resting-state fMRI, gradient EPI sequence (3.8 mm slice thickness, TR 2 s/TE 30 ms, FOV 24 cm, 64 × 64 matrix, 7:08 min scan time). Cardiac and respiratory waveforms were collected independently during the EPI scans for later removal; 3) multi-slice diffusion weighted imaging was acquired using a single-shot spin-echo echo-planar sequence with 64 contiguous axial slices (slice thickness = 2.5 mm, FOV = 240 × 240 mm). Diffusion weighting was performed with 80 non-collinear directions with multiple b values: b = 0 s/mm^2^, b = 300 s/mm^2^ and b = 1100 s/mm^2^; 4) axial T2-weighted images were also acquired for EPI distortion correction purpose, with a fast spin echo sequence with the same FOV and 1.7 mm slice thickness.

### 2.3 Functional MRI

Preprocessing was done with the AFNI software package (Cox, 1996) using the basic approach described by Gotts and colleagues (Gotts et al., 2012; Gotts et al., 2013). Briefly, the first 4 EPI volumes were removed AFNI 3dDespike was used to remove large transients (due to factors such as head movement (Jo et al., 2013)). Time series were corrected for slice-time acquisition, and all EPI volumes were co-registered with the T1-weighted scan to the first volume in the truncated set, then spatially blurred by a 6-mm (full width at half maximum) Gaussian kernel, with each voxel's time series normalized by its temporal mean to yield units of percent signal change. Linear regression was then used to remove motion (6 motion parameters), cardiac and respiratory cycles (8 regressors for slice time 0;(Glover et al., 2000)), and slower effects of respiration (5 respiration volume per time regressors;(Birn et al., 2008)), as well as average signal from the ventricles and a local average of white matter signal (within a radius of 20 mm centered on each voxel) calculated prior to the spatial blurring step. Ventricle and white matter time series were derived for each subject by segmenting the T1 weighted scan into gray, white, and CSF compartments using FreeSurfer (Fischl et al., 2002), and these masks were resampled to EPI resolution and eroded by 1 voxel to prevent partial volume effects with gray matter. With the exception of the cardiac/respiration regressors (which already incorporate time delays), delayed versions (1 TR) of all regressors were included to allow for delayed effects of noise sources. The cleaned, blurred residual time series were then spatially normalized to the MNI anatomical template (http://www.bic.mni.mcgill.ca/ServicesAtlases/Colin27, as implemented in AFNI's MNI_caez_N27 template) for the purposes of group analyses using each subject's anatomical scan. An index of transient head motion (AFNI's @1dDiffMag) was calculated from each subject's motion parameters for use as a nuisance covariate in the group-level analyses.

#### 2.3.1 Functional connectivity maps

**Fig. 1:**
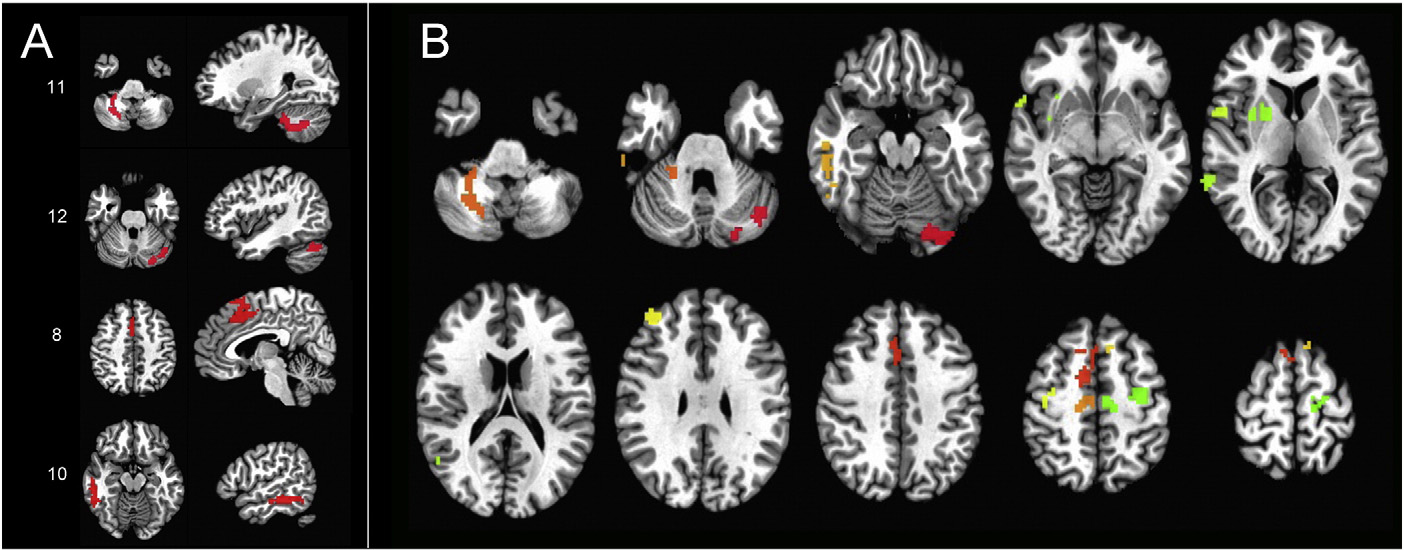
Regions with increased connectivity in PLS patients compared to healthy controls. A) “Seed” regions used to find connected ROIs with increased connectivity. Seeds are shown on axial (left) and sagittal (right) projections. Numbering corresponds to ROI position in the correlation matrix: 11. Right cerebellar seed, 12. Left cerebellar seed, 8. Cingulate seed,10. Right middle inferior temporal seed. B) Twelve ROIs, including original 4 seeds, with increased connectivity in PLS patients projected on axial sections. Sections shown in radiological convention with the right brain on the left side.

Functional connectivity was assessed for each participant in a whole-brain fashion (Gotts et al., 2012). The average Pearson correlation of each voxel's time series with all voxels in a whole-brain mask was first calculated using AFNI's 3dTcorrMap function. These average correlations were then transformed using Fisher's z to yield normally distributed values and then compared in MNI coordinates between PLS patients and controls using a basic ANCOVA approach (AFNI's 3dttest++), covarying the level of transient head motion for each participant (see Saad et al., 2013 for discussion). Four clusters of voxels survived cluster-size correction for whole-brain comparisons at P < 0.05 (voxel-wise P-value threshold of P < 0.005) and served as seeds in the next step of the analyses (Fig. 1A). The four clusters were tested individually in more standard seed-based correlation analyses between PLS and control groups using 3dttest++ (covarying transient head motion), correcting for whole-brain comparisons using both cluster size and the number of seeds tested (corrected to P < 0.05/4 = 0.0125, with an initial voxel-wise P-value threshold of P < 0.005). Following the seed tests, additional voxels were included in subsequent ROI analyses if they were present in the corrected group comparisons of all 4 seeds, yielding an additional 8 voxel clusters for a total of 12 ROIs (see Fig. 1B for details).

**Fig. 2:**
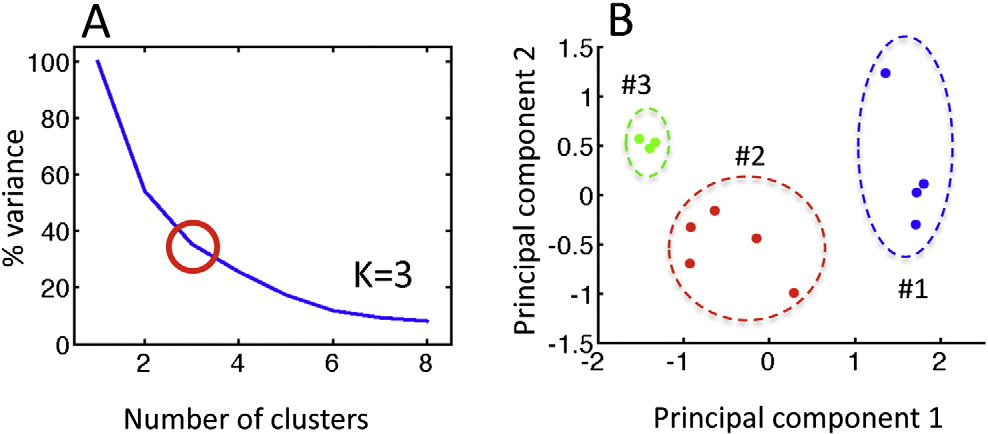
K-means cluster analysis of functionally connected regions of interest. A) Elbow plot, showing 3 clusters is the best trade-off of variance explained to model complexity. B) Principle components analysis of the 12 × 12 ROI–ROI correlation matrix (averaged across groups); cluster membership is depicted simultaneously using color (green, red, blue). Cluster labeled #3, shown in green, consisted left precentral/SMA, right precentral, and right SMA ROIs; cluster in red labeled #2 consisted of right cingulate, right mid-frontal, right superior temporal, right mid-temporal, right putamen ROIs; cluster in blue labeled #1 consisted of left cerebellum, right cerebellum, right mid-temporal ROIs (SMA = supplementary motor area).

**Fig. 3:**
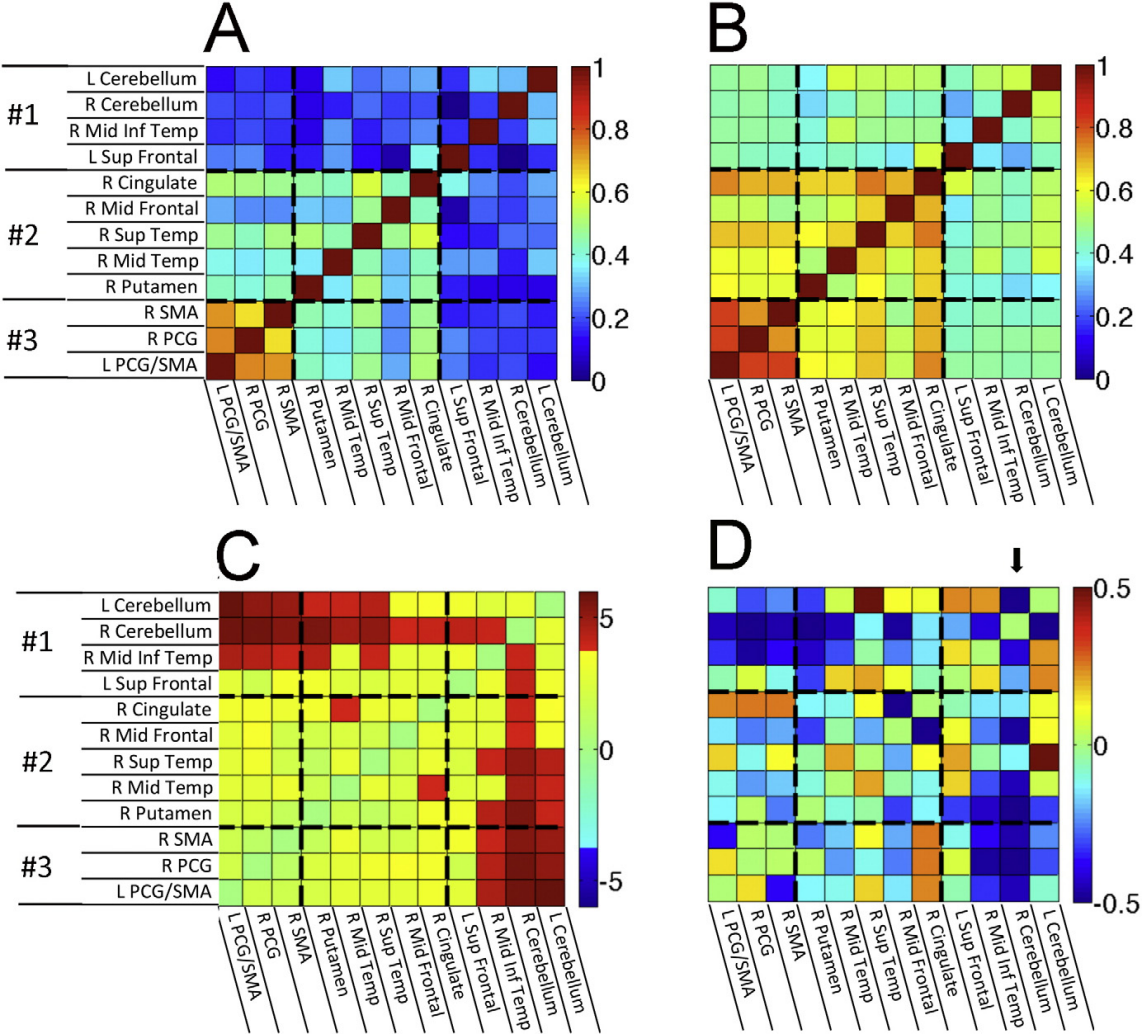
Correlation matrices of the BOLD signal between 12 ROIs showing increased functional connectivity in A) healthy controls and B) PLS patients. Scale bar indicates correlation with warmer colors for more strongly correlated regions. C) Matrix showing ROIs in which the correlations differ between PLS patients and controls. Warmer colors indicate stronger correlations in PLS patients. The scale bar indicates T values. D) Matrix showing the correlation between the ALSFRS-R and connectivity of ROIs with the right cerebellar seed. Scale bar indicates the partial correlation coefficient. The arrow points to the column showing the correlation between the ALSFRS-R and connectivity of ROIs to the right cerebellar seed.

All-to-all ROI correlation matrices were calculated for each participant using the average voxel time series from each of the 12 ROIs, transforming the resulting correlations using Fisher's z. The structure of the group-average correlation matrix (pooling both groups) was then analyzed using K-means cluster analyses, principal components analysis (PCA, viewing 1st two PCs), and multi-dimensional scaling (MDS), with an “elbow” criterion on the tradeoff of variance explained versus model complexity yielding 3 clusters and good agreement across the different analysis methods (Fig. 2). Results for single-group correlation matrices, group comparisons, and behavioral correlations for the PLS group were then viewed after sorting ROIs by cluster membership (Fig. 3). As with the other group comparisons of correlation magnitude, values were corrected for the number of comparisons to P < 0.05 (Bonferroni) after covarying age and transient head motion values.

**Fig. 4:**
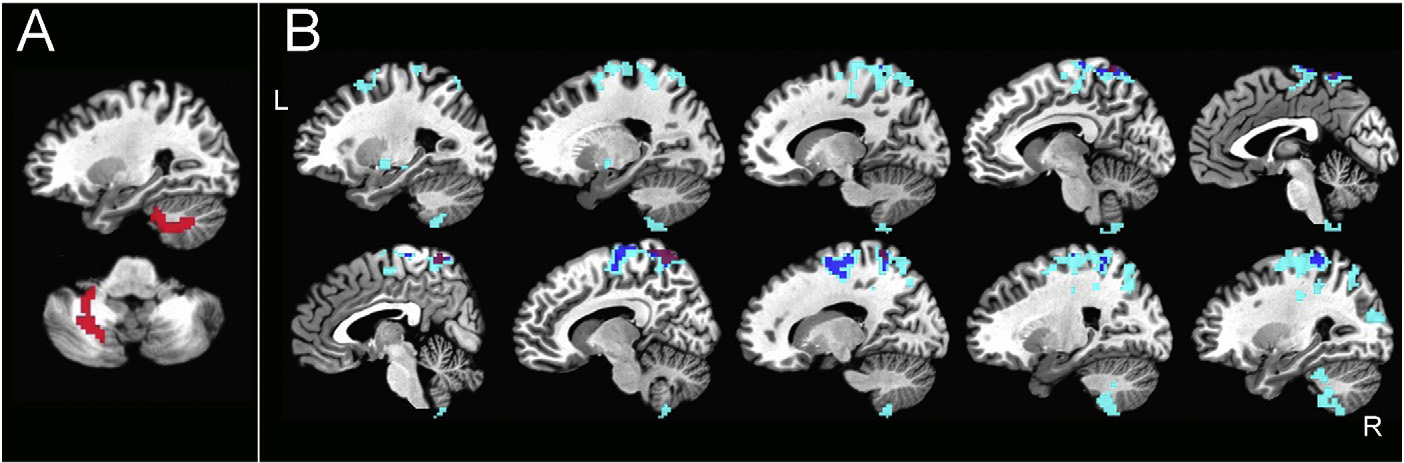
Brain regions showing correlation between ALSFRS-R score and connectivity with right cerebellar seed. A) Right cerebellar seed, B) regions with significant correlation of ALSFRS-R with cerebellar connectivity. Colors indicate P value. Light blue = P < 0.05, dark blue = P < 0.01, purple = P < 0.005. Sagittal slices shown from right (top row) to left (bottom row).

Correlations of functional connectivity measures with clinical symptoms for the PLS patients were carried out for ROI-level and whole-brain data using partial correlation to remove the effects of transient motion and age. For the whole-brain analyses, correlation maps for each PLS patient were calculated from each of the four seeds detected in the group comparisons and transformed using Fisher's z. Partial correlations with ALSFRS-R score were then carried out across patients, partialing transient motion and age and correcting for whole-brain comparisons using both cluster size and number of seeds tested (to P < .05/4) over a range of voxel-wise P-value thresholds (P < 0.05, P < 0.01, and P < 0.005; see Fig. 4). Correlation of functional connectivity measures with disease duration and progression rate (calculated as (48 − ALSFRS-R score) / duration in months) was explored with Spearman's correlation using P < 0.05 (uncorrected) as threshold.

### 2.4 Diffusion tensor imaging

Algorithms in the TORTOISE software package (Pierpaoli et al., 2010) were used for processing raw diffusion-weighted images, including correcting motion artifacts, eddy current distortion, and calculation of the tensor. The DTI metric maps were derived in TORTOISE.

#### 2.4.1 Tract based spatial statistics

To visualize differences in white matter integrity between healthy controls and PLS patients, a voxel-wise statistical analysis of the FA skeletons was carried out using Tract-based spatial statistics (TBSS v1.2, http://www.fmrib.ox.ac.uk/fsl/ (Andersson, Jenkinson, and Smith 2007a, Andersson, Jenkinson, and Smith 2007b; Rueckert et al., 1999; Smith et al., 2004)). The Randomize tool in FSL (v2.1, 5000 permutations), (Nichols and Holmes, 2002), which conducts permutation-based inference on t-statistic maps, was used to identify clusters of voxels that differed between controls and the patient group, using the TFCE (Threshold-Free Cluster Enhancement) with the threshold for significance of P < 0.05 with corrections for multiple comparisons across space (Family wise Error Rate, FWE), with age as a covariate.

#### 2.4.2 Structural connectivity between functionally connected regions

Each subject's raw DTI data were registered to standard space (MNI 152 2 × 2 × 2 mm) using FLIRT (FMRIB's Linear Image Registration Tool) to perform linear registration between subject's b0 and the T1-weighted image, and FLIRT to register the T1-weighted images to standard space (Jenkinson et al., 2012). Probabilistic fiber tracking was carried out using Probtrackx2 (Behrens et al., 2007) to characterize the connectivity distribution between the 12 ROIs identified in the resting state fMRI study. Each ROI was used as a seed region with 5000 streamlines generated at each voxel and a curvature threshold of 0.2. A 12 × 12 connectivity matrix was constructed for each subject showing the probability of a streamline between pairs of ROIs. Connectivity measures were normalized according to the size of the seed and the target region. Finally, because directionality cannot be determined by tractography, the values for each seed-target pair were averaged across both directions to produce a symmetric matrix (Owen et al., 2013).

### 2.5 Statistics

The demographic data and clinical measures are shown as mean and standard deviations, and differences between groups were assessed with two-sample t-tests, using a corrected (FWE) threshold of P < 0.05 for significance. For group comparisons of EPI data, robustness to transient head motion was established through the application of ANCOVA rather than simple t-tests, using motion and age as covariates as described above (AFNI's 3dttest++). Correlation of clinical measures with rs-fMRI ROI–ROI correlation values across PLS patients utilized partial correlation coefficients, covarying transient motion and age, with results for the full ROI–ROI matrix shown un-thresholded for comparison with the other matrices. Correlation of clinical measures using seed-based correlation maps for individual PLS patients, also partialing transient motion and age, was corrected for whole-brain comparisons using cluster size and number of seeds tested to P < 0.05. Wilcoxon rank sum test was used to compare group medians of the ROI–ROI tractography correlation matrices between PLS patients and controls.

## 3 Results

### 3.1 Clinical features

Sixteen PLS patients (12 men, 4 women; mean age 59.7 ± 8.6 years) and 14 healthy controls (12 men, 2 women; mean age 51.6 ± 10.3 years) participated in the study (Table 1). There was no difference in gender ratios between the groups. PLS patients had significantly slower finger and foot tapping rates compared to controls. There was no difference between the groups on the mini-mental state examination. Resting state functional MRI studies were obtained on all subjects. The DTI data were adequate for structural connectivity analysis in 15 PLS patients and 13 healthy controls.

### 3.2 Resting-state fMRI

There were no regions with decreased functional connectivity in PLS patients compared to controls. Four regions that had increased functional connectivity with the rest of the brain were identified in PLS patients compared to controls. These regions were located in the right and left cerebellum, the right middle cingulate gyrus, and the right middle/inferior temporal gyrus (Fig. 1A). These four “seeds” were used to identify other functionally connected regions. Eight additional regions were identified that had increased connectivity with all 4 seed regions in patients (FWE corrected to P < 0.05 for whole-brain comparisons and 4 seeds tested).

The correlation matrices show the pairs of ROIs with strongly correlated resting brain activity in PLS patients (Fig. 3B) and controls (Fig. 3A). Activity in the bilateral precentral gyri and supplementary motor areas was strongly correlated in both patient and control groups (ROIs in cluster 3). However, several other ROI pairs had significantly stronger correlations in PLS patients than controls (Bonferroni-corrected pairs shown in red in Fig. 3C).

Inter-relationships of all ROI–ROI pairs were examined using K-means cluster analysis, PCA (1st two principal components), and multi-dimensional scaling (reducing 12 ROI dimensions to 2 dimensions), yielding corresponding results for 3 main clusters of ROIs (Fig. 2). There was increased functional connectivity between the two cerebellar ROIs and the right inferior temporal region in cluster 1 with the precentral gyrus and SMA bilaterally in cluster 3. There was also increased connectivity between the two cerebellar ROIs of cluster 1 with the right putamen, right middle temporal, right superior temporal ROIs of cluster 2, and the right cerebellum with the right middle frontal and right cingulate ROIs of cluster 2.

**Fig. S1:**
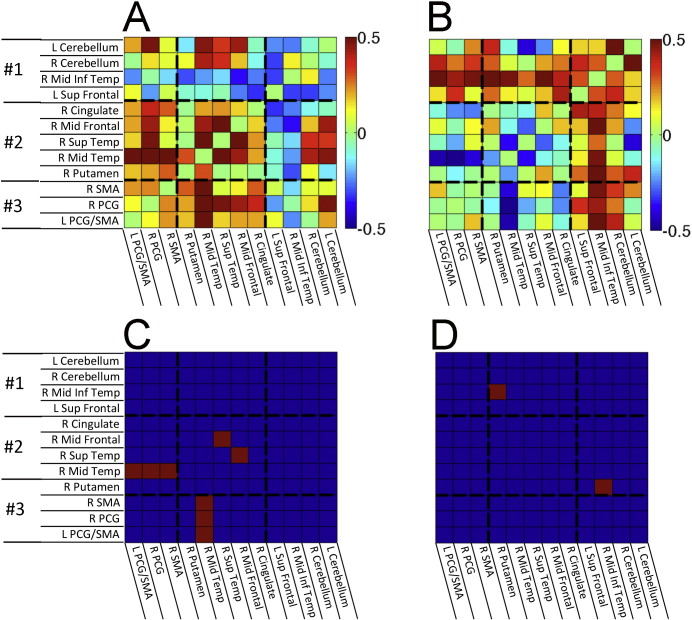
Correlation of increased functional connectivity between the 12 ROIs in PLS patients with A) disease duration and B) progression rate. Scale bar indicates Spearman correlation coefficient with warmer colors for more strongly and positively correlated regions. C) Red ROI pairs are those in which the significance of the correlation of increased functional connectivity with disease duration was <0.05 (uncorrected). Increased connectivity of cortical motor regions (cluster 3) with the right mid-temporal region was correlated with increased disease duration. D) The red ROI pair between two temporal regions is the only one in which the significance of the correlation of increased functional connectivity with progression was <0.05 (uncorrected).

Of the 12 ROIs, increased connectivity was greatest between the right cerebellar ROI and most other ROIs. The strength of connectivity between the right cerebellar ROI–other ROIs was inversely correlated with the ALSFRS-R of the PLS patients, i.e. greater functional connectivity values were associated with smaller ALSFRS-R scores and greater clinical impairment (Fig. 3D). Regions showing a correlation between right cerebellar connectivity and ALSFRS-R are shown in Fig. 4, with the largest negative correlations observed in the right sensory and motor regions. Disease duration was not correlated with increased functional connectivity between cerebellar ROIs of cluster 1 and other regions, but was significantly correlated with increased connectivity between cluster 3 motor cortex ROIs and the right mid-temporal ROI of cluster 2 (Supplementary Fig. 1A, B). This pattern differed from the correlation between disease severity, as measured by the ALSFRS-R and connectivity. Progression rate, calculated from the ALSFRS-R score and disease duration, showed similar directions of correlation between functional connectivity as these measures individually, which mostly did not reach significance (Supplementary Fig. 1C, D).

Inline Supplementary Figure S1

### 3.3 Diffusion tensor imaging

#### 3.3.1 Tract based spatial statistics

**Fig. 5:**
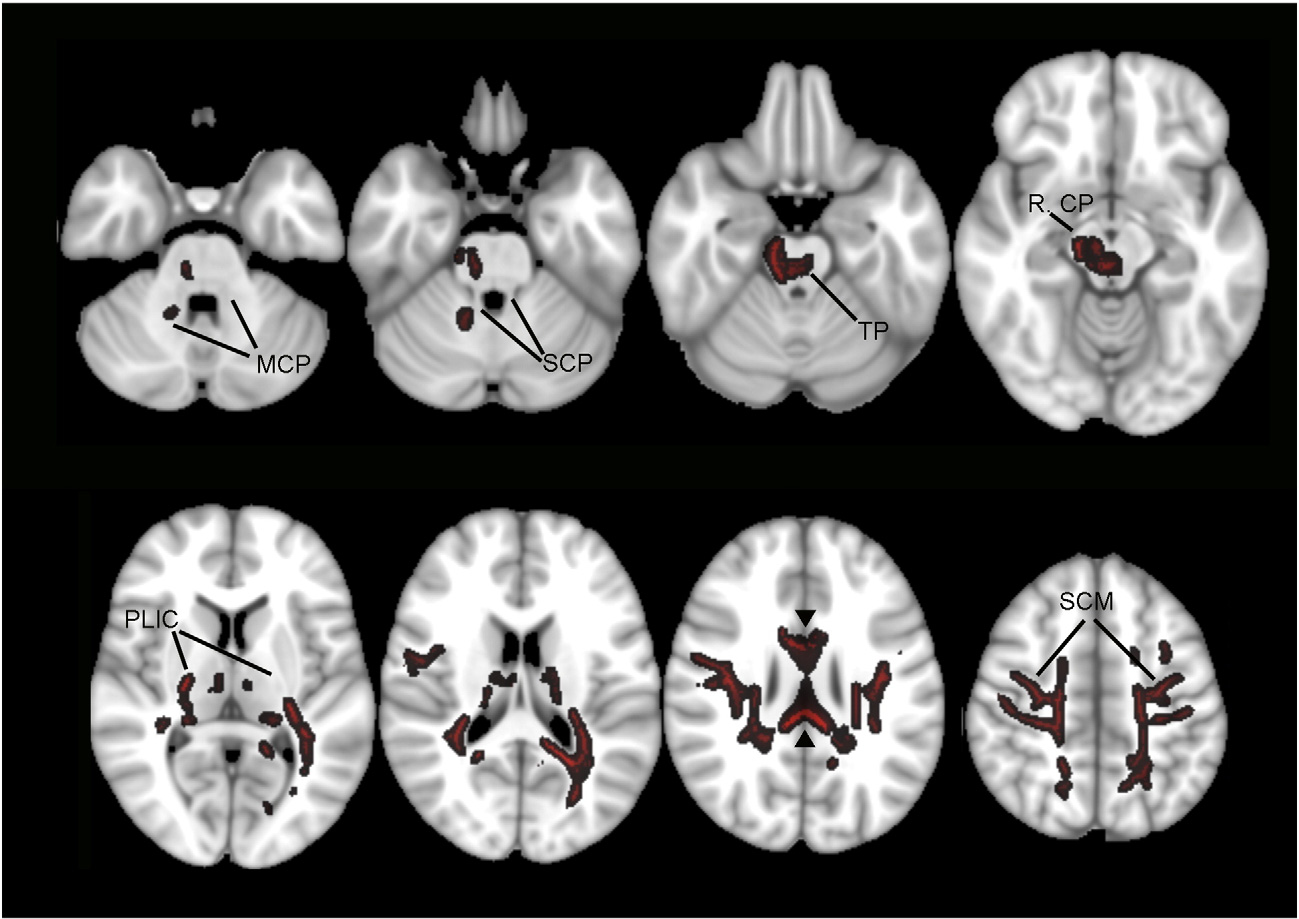
White matter regions with reduced fractional anisotropy in PLS patients compared to controls are shown in red. TBSS analysis (P < 0.05, TFCE with FWE correction for multiple connection). Axial sections are shown in radiological convention with right on the left side. MCP, middle cerebellar peduncle; SCP, superior cerebellar peduncle; RCP, right cerebral peduncle; TP, transverse pontine fibers; PLIC, posterior limb of the internal capsule; SCM, subcortical white matter. Arrowheads point to the corpus callosum.

There was widespread loss of fractional anisotropy of the cortical white matter in the PLS patient group compared to controls in the TBSS analysis. The changes were prominent in the subcortical white matter underlying the pre- and post-central gyrus, extending into the posterior limb of the internal capsule and right cerebral peduncle, as well as the corpus callosum (Fig. 5). Of note, although fractional anisotropy was reduced in the transverse pontine fibers and right middle cerebellar peduncle, the superior cerebellar peduncles – cerebellar outflow tracts – were unaffected.

#### 3.3.2 Structural connectivity

**Fig. 6:**
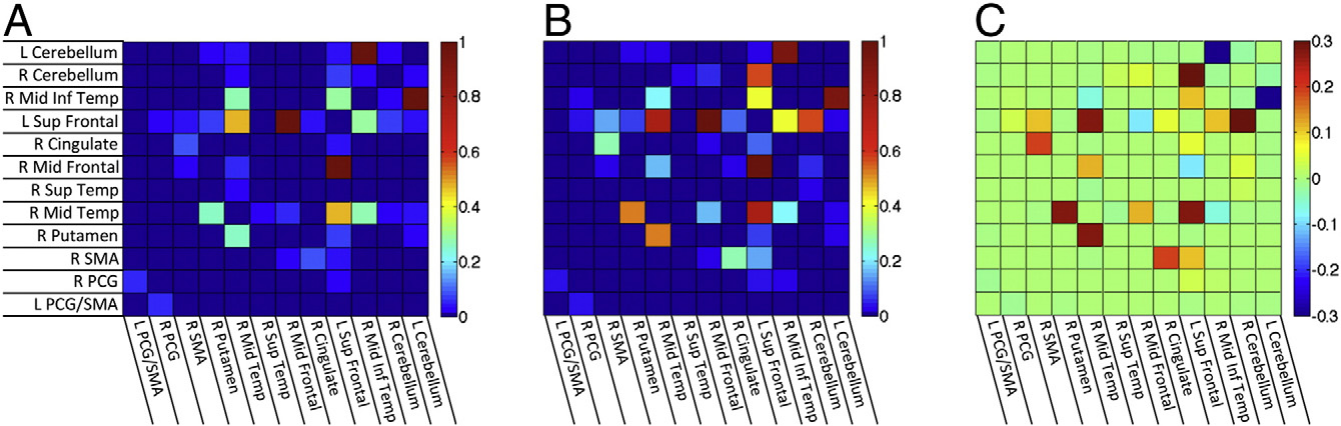
Results of probabilistic tractography using each of the 12 ROIs as a seed, with results expressed in terms of the probability of a connection. A) Median connection probability for each ROI × ROI combination across control subjects, and B) for PLS patients. Group differences are shown in C) as the PLS median−control median. No group differences survived correction for multiple comparisons, and the pattern of the results appeared unrelated to the group differences in functional connectivity (compared to Fig. 2).

Structural connections between the ROIs showing increased functional connectivity in PLS were visualized with probabilistic tractography. The group matrices showing the probability of structural connections between pairs of ROIs were highly similar (Spearman's r = 0.934, R^2^ = 0.873) for controls (Fig. 6A) and PLS patients (Fig. 6B), and no group differences survived correction for multiple comparisons. Furthermore, the group differences in tractography bore little resemblance to the group differences in functional connectivity (compare to Fig. 2). Quantitatively, the group difference matrices (Fig. 2, Fig. 6) shared almost no variance in common (Spearman's r = 0.008, R^2^ = 0.007), suggesting that structural connectivity differences are unlikely to explain the pattern of functional connectivity differences between the PLS and control groups.

## 4.0 Discussion

Patients with primary lateral sclerosis exhibited increased functional connectivity in resting-state fMRI scans, in agreement with several other recent studies of patients with motor neuron disorders (Agosta et al., 2013; Douaud et al., 2011; Jelsone-Swain et al., 2010; Verstraete et al., 2010). A distinct finding from our study, however, was the predominance of increased cerebro-cerebellar functional connectivity, which was stronger than the smaller increase in sensorimotor connectivity that was also observed. The identification of the cerebellum in the functional network resulted from our data-driven approach to identify areas that differed most from controls, and examining functional and structural connections between the identified regions. This approach identified certain regions residing within previously described regional resting-state networks in healthy subjects, such as the precentral gyrus and supplementary motor area in the sensorimotor network, as well as additional regions, such as the cerebellum and putamen. The association between increased cerebro-cerebellar functional connectivity to poorer motor functioning, as measured by the ALSFRS-R score, within the PLS group demonstrates the relevance of this phenomenon: the increased cerebro-cerebellar correlations in PLS cannot be dismissed easily as epiphenomenal or due to co-morbidities. Disease duration was not correlated with the increased cerebro-cerebellar connectivity, although this should be interpreted with caution since changes within the first 3 years would not be captured.

The finding of increased functional connectivity in motor networks in PLS and ALS is quite distinct from Alzheimer disease, in which resting state studies consistently show disconnection of cognitive brain networks subserving cognition (Brier et al., 2014; Gour et al., 2014; Li and Wahlund, 2011). Clinical symptoms in Alzheimer disease have been correlated with progressive loss of both functional and structural connectivity as neurodegeneration proceeds (Hahn et al., 2013). Functional disconnection of particular networks also occurs in non-degenerative neuropsychiatric disorders, such as autism and schizophrenia (Alexander-Bloch et al., 2013; Gotts et al., 2012).

Despite the increased functional connectivity in PLS patients, no difference was seen in structural connections between regions with increased functional connectivity. Diffusion tensor imaging confirmed that fractional anisotropy was reduced in the corticospinal tract, the major long-range outflow tract from the motor cortex, and the corpus callosum, indicative of axonal disruption (Basser and Pierpaoli, 1996; Beaulieu, 2002). By contrast, the cerebellum's major outflow tract, the superior cerebellar peduncle, showed no changes in diffusion measures from controls. These findings suggest that the increased functional connectivity does not depend on direct axonal connections between regions, or growth of new axonal connections. The mechanism underlying this paradoxical increase of functional connectivity in the face of reduced structural integrity of white matter tracts is uncertain. Functional connectivity has a complex relationship to structural connectivity. When structural connections between regions are present, the strength of those connections predicts the strength of functional connectivity, but the converse does not hold true (Damoiseaux and Greicius, 2009; Honey et al., 2009). In some cases, functional connectivity between regions may result from common inputs (Ansari et al., 2011). One possibility is that the correlated functional activity in cerebellum and cortical regions was generated indirectly through common inputs. Structural connections over both short and long paths shape functional connectivity (Alexander-Bloch et al., 2013; Honey et al., 2010; van den Heuvel et al., 2009). Altered functional connectivity could reflect differences in the utilization of multisynaptic short-range connections in brain networks versus the long-range connections that are better visualized by DTI tractography.

Changes in functional connectivity could also result from an alteration of the balance between local excitatory and inhibitory interneurons due to dysfunction of inhibitory interneurons in the cortex. Pathological studies have found that certain classes of GABAergic interneurons undergo degeneration in ALS (Maekawa et al., 2004), lending support to the hypothesis that loss of inhibitory tone contributes to clinical dysfunction in ALS (Turner and Kiernan, 2012). Physiological studies and flumazenil PET studies also provide evidence for loss of intracortical inhibition in ALS (Enterzari-Taher et al., 1997; Turner and Kiernan, 2012; Wittstock et al., 2007). Intracortical inhibitory circuits normally enhance selective activation of cortical representations for specific movements while inhibiting surrounding representations for undesired movements (Beck and Hallett, 2011). The loss of inhibition, in the setting of intact cortical afferents, would result in larger regions of cortical activation but impairment of fine motor control.

An alternative view of increased functional connectivity is that it represents a form of plasticity, with recruitment of additional brain regions or redundant pathways that compensate for the loss of corticospinal neurons (Tessitore et al., 2006). This idea is supported by task-based fMRI studies showing recruitment of extramotor and subcortical regions in ALS, including the cerebellum (Konrad et al., 2002; Lule et al., 2009). Similar expanded activation also occurred with imagined movements (Poujois et al., 2013). However, in ALS, task-based activation of extramotor areas declined with progressive weakness, indicating that recruitment of extramotor cortex could only provide transient compensation (Mohammadi et al., 2011). Given the reduced integrity of corticofugal tracts, it seems unlikely that new axonal connections account for increased connectivity in PLS patients.

Although pathological studies have shown ubiquinated inclusions in the cerebellum, particularly in familial forms of ALS (Hsiung et al., 2012; Prell and Grosskreutz, 2013), there is little clinical evidence for cerebellar dysfunction in PLS. The patients in this study did not have clinical signs typical of cerebellar motor dysfunction such as ataxia, nystagmus, or dysmetria. Cerebellar–cerebral connectivity has been shown to be disrupted in disorders such as Freidreich's ataxia that do manifest such cerebellar signs (Zalesky et al., 2014). In PLS, the increased functional connectivity with the cerebellum reflects the relative integrity of cerebellar outflow tracts, with increased reliance on proper cerebellar activity to maintain motor coordination.

## Figures and Tables

**Table 1:** Demographic and clinical features of study participants.

	Controls n = 14	PLS patients n = 16	P value
Male:female	12:2	12:4	
Age (yrs)	51.6 ± 10.3	59.7 ± 8.6	0.027
Disease duration (mos)		104 ± 48	
ALSFRS-R		35.8 ± 5.8	
Finger taps/s (R)	6.2 ± 0.9	3.4 ± 1.1	<0.001
Finger taps/s (L)	6.1 ± 1.1	2.7 ± 0.8	<0.001
Foot taps/s (R)	4.5 ± 0.6	1.8 ± 0.6	<0.001
Foot taps/s (L)	4.1 ± 0.8	1.5 ± 0.6	<0.001
Mini-mental state examination	29.0 ± 1.2	29.3 ± 1.6	0.636
